# Inhibition of the MAP2K7-JNK pathway with 5Z-7-oxozeaenol induces apoptosis in T-cell acute lymphoblastic leukemia

**DOI:** 10.18632/oncotarget.28040

**Published:** 2021-08-31

**Authors:** Taylor J. Chen, Wa Du, Jacob J. Junco, Cory Seth Bridges, Ye Shen, Monica Puppi, Karen R. Rabin, H. Daniel Lacorazza

**Affiliations:** ^1^Department of Pathology & Immunology, Baylor College of Medicine, Houston, TX, USA; ^2^Texas Children’s Cancer Center, Department of Pediatrics, Baylor College of Medicine, Houston, TX, USA

**Keywords:** T-ALL, MAP2K7, 5z-7-oxozeaenol

## Abstract

T-cell acute lymphoblastic leukemia (T-ALL) is an aggressive pediatric leukemia with a worse prognosis than most frequent B-cell ALL due to a high incidence of treatment failures and relapse. Our previous work showed that loss of the pioneer factor KLF4 in a NOTCH1-induced T-ALL mouse model accelerated the development of leukemia through expansion of leukemia-initiating cells and activation of the MAP2K7 pathway. Similarly, epigenetic silencing of the *KLF4* gene in children with T-ALL was associated with MAP2K7 activation. Here, we showed the small molecule 5Z-7-oxozeaenol (5Z7O) induces dose-dependent cytotoxicity in a panel of T-ALL cell lines mainly through inhibition of the MAP2K7-JNK pathway, which further validates MAP2K7 as a therapeutic target. Mechanistically, 5Z7O-mediated apoptosis was caused by the downregulation of regulators of the G2/M checkpoint and the inhibition of survival pathways. The anti-leukemic capacity of 5Z7O was evaluated using leukemic cells from two mouse models of T-ALL and patient-derived xenograft cells generated using lymphoblasts from pediatric T-ALL patients. Finally, a combination of 5Z7O with dexamethasone, a drug used in frontline therapy, showed synergistic induction of cytotoxicity. In sum, we report here that MAP2K7 inhibition thwarts survival mechanisms in T-ALL cells and warrants future pre-clinical studies for high-risk and relapsed patients.

## INTRODUCTION

Acute lymphoblastic leukemia (ALL) is the most common pediatric cancer, with more than 3,000 new cases diagnosed every year in the U.S. [1, 2]. While advances in pediatric ALL therapies through systemic and intrathecal multi-drug treatment have vastly improved 5-year event-free survival over 85% [3], relapsed ALL patients exhibit poor prognosis and remain the leading cause of pediatric cancer-related mortality [4–7]. ALL is broadly classified based on immunophenotype into B-cell or T-cell ALL, the latter being less frequent albeit with worse prognosis and higher rate of relapse. Relapses after induction therapy or following remission are caused by the outgrowth of drug-resistant leukemic cells with leukemia initiating capacity. In order to provide therapeutic options for patients with refractory disease, it is essential to identify novel targets for the development of alternative therapies.

The mitogen-activated kinase kinase MAP2K7 is an emerging target for anticancer drug therapy [8]. Our group described that the pioneer factor KLF4 inhibits homeostatic and antigen-driven proliferation of T cells and that genetic inactivation of KLF4 associates with more aggressive leukemia in NOTCH1-induced T-ALL mouse models and children with T-ALL by aberrantly activating the MAP2K7 signaling pathway [9–12]. This pathway is a three-tiered mitogen-activated protein kinase signaling composed by MAP3K, MAP2K (MAP2K7), and the c-jun N-terminal kinase (JNK) [13]. In response to stress signals, MAP3K activates MAP2K7 through phosphorylation at serine and threonine residues, which in turn activates JNK and downstream effectors like c-JUN and ATF2 [13, 14]. A loss-of-function study shows MAP2K7 mediates stress-induced JNK activation in mast cells and inhibits growth factor and antigen-driven proliferation of immune cells [15].

Pharmacological inhibition of JNK, the sole downstream substrate of MAP2K7, with the selective JNK inhibitor JNK-IN-8 and other drugs tested in clinical trials showed anti-leukemic properties in T-ALL, validating the MAP2K7-JNK pathway as a therapeutic target. However, JNK inhibition presents important caveats for clinical translation regarding low potency and off-target effects [9]. To overcome these limitations, we propose the direct inhibition of the MAP2K7 kinase as a novel therapeutic approach for T-ALL. Generation of small molecules able to selectively inhibit MAP2K7 has proven difficult in light of the prevalence of structural homology within the MAP2K family of proteins, especially in the ATP binding pocket [8]. Selective inhibition of MAP2K7 would reduce pleiotropic effects because MAP2K4 activates both p38 and JNK, whereas MAP2K7 only activates JNK, but a challenge is the functional and structural similarities between MAP2K4 and MAP2K7, the only known kinases able to activate JNK [16]. Analysis of the ATP binding pocket across the MAP2K family of proteins revealed a cysteine-218 located in the hinge region of MAP2K7 that is not present in other MAP2K proteins, offering a window of opportunity for irreversible and selective inhibition [8, 17]. In fact, the compound 5Z-7-oxozeaenol (5Z7O) possesses the unique ability to bind to the cysteine-218 in MAP2K7, although it can also inhibit other kinases such as TAK1 [18]. Overall, these data support MAP2K7 as a new therapeutic target in T-ALL and that pharmacological inhibition with small molecules may have therapeutic potential.

We report here the anti-leukemic properties of 5Z7O in T-ALL and show dose-dependent cytotoxicity in a panel of T-ALL cell lines through inhibition of MAP2K7. Mechanistically, 5Z7O induced DNA damage, inhibited cell proliferation and accumulated cells in G0 phase of the cell cycle, deregulated the G2/M checkpoint, and induced apoptosis. Further, 5Z7O selectively targets leukemic T cells in two T-ALL mouse models and patient-derived xenograft cells and shows synergism when combined with dexamethasone. These findings underscore the therapeutic potential of MAP2K7 inhibition for the treatment of T-ALL.

## RESULTS

### T-ALL cell line response to MAP2K7 inhibition with 5Z-7-oxozeaenol

**Figure 1:**
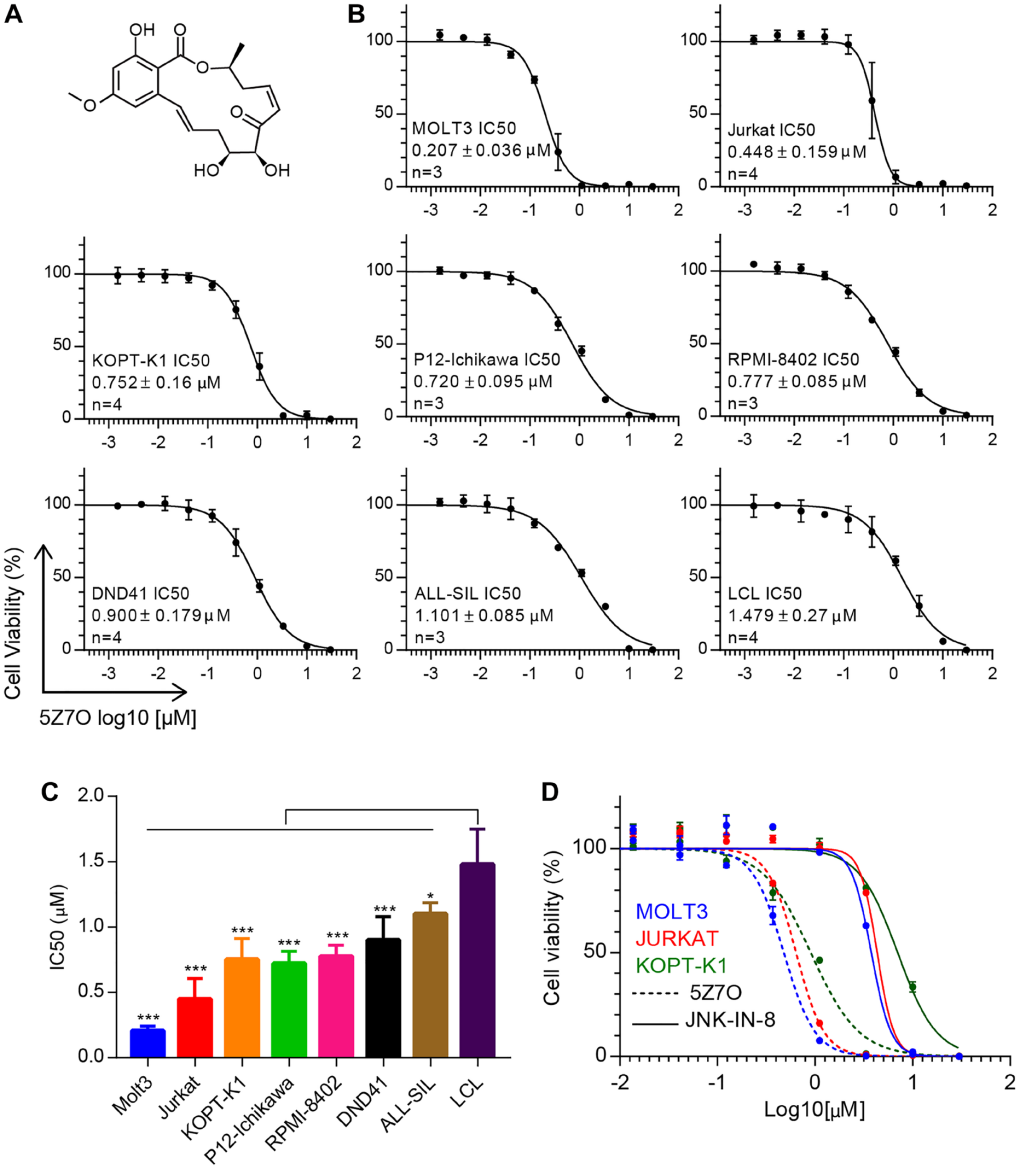
5Z-7-oxozeaenol (5Z7O) inhibits cell viability in T-ALL cell lines. (**A**) Chemical structure for 5Z7O. (**B**) Cell viability assays in T-ALL and LCL cell lines treated with 5Z7O for 48 hours (*n* = 3). Cell viability was measured using an ATP-based cell viability assay and expressed as a percentage of the vehicle (DMSO) control. (**C**) IC50 values were calculated using GraphPad for 48 hours incubation. The data represent the mean and standard deviation (*n* = 4). (**D**) Cell viability assays comparing 5Z7O and JNK-IN-8 compounds in Molt3, Jurkat, and KOPT-K1 T-ALL cell lines cultured for 48 hours were measured using an ATP-based cell viability assay and expressed as a percentage of the vehicle control (DMSO). Cell viability assays were averaged from 3–4 independent experiments. ^*^ *P* < 0.05, ^**^ *P* < 0.01, ^***^ *P* < 0.001; two-tailed Student’s *t*-test.

The chemical compound 5Z-7-oxozeaenol (5Z7O, Figure 1A) has been described to bind a free cysteine residue located in the hinge region of the ATP binding pocket in MAP2K7 through covalent reaction (Supplementary Figure 1) [18]. Based on our previous findings that patients with T-ALL exhibit aberrant activation of MAP2K7 [9], we decided to investigate the anti-leukemic properties of MAP2K7 inhibition with 5Z7O in a panel of T-ALL cell lines using an EBV-transformed lymphoblastoid cell line (LCL) as a non-leukemic control. We found that 5Z7O treatment for 48 hours reduced cell viability in a dose-dependent manner with IC50 values ranging from 200 nM for Molt3 to 1.1 μM for ALL-SIL, while LCL cells displayed an IC50 of 1.5 μM (Figure 1B). Therefore, most T-ALL cell lines possessed significantly higher sensitivity compared to LCL cells (Figure 1C). These data suggest that 5Z7O inhibits leukemic cell viability either by inhibiting cell proliferation or inducing apoptosis and that some T-ALL cell lines (e.g., Molt3, KOPT-K1, and Jurkat) were more sensitive to 5Z7O. Furthermore, we directly compared the anti-leukemic cytotoxicity of 5Z7O with the previously described JNK inhibitor (JNK-IN-8) [19]. Molt3, Jurkat, and KOPT-K1 cell lines were more sensitive to 5Z7O compared to JNK-IN-8, with an IC50 7-fold lower (Figure 1D). This finding suggests that MAP2K7 inhibition with 5Z7O may be more effective than JNK inhibition in T-ALL.

### 5Z7O inhibits cell proliferation and induces apoptosis in T-ALL cells

**Figure 2:**
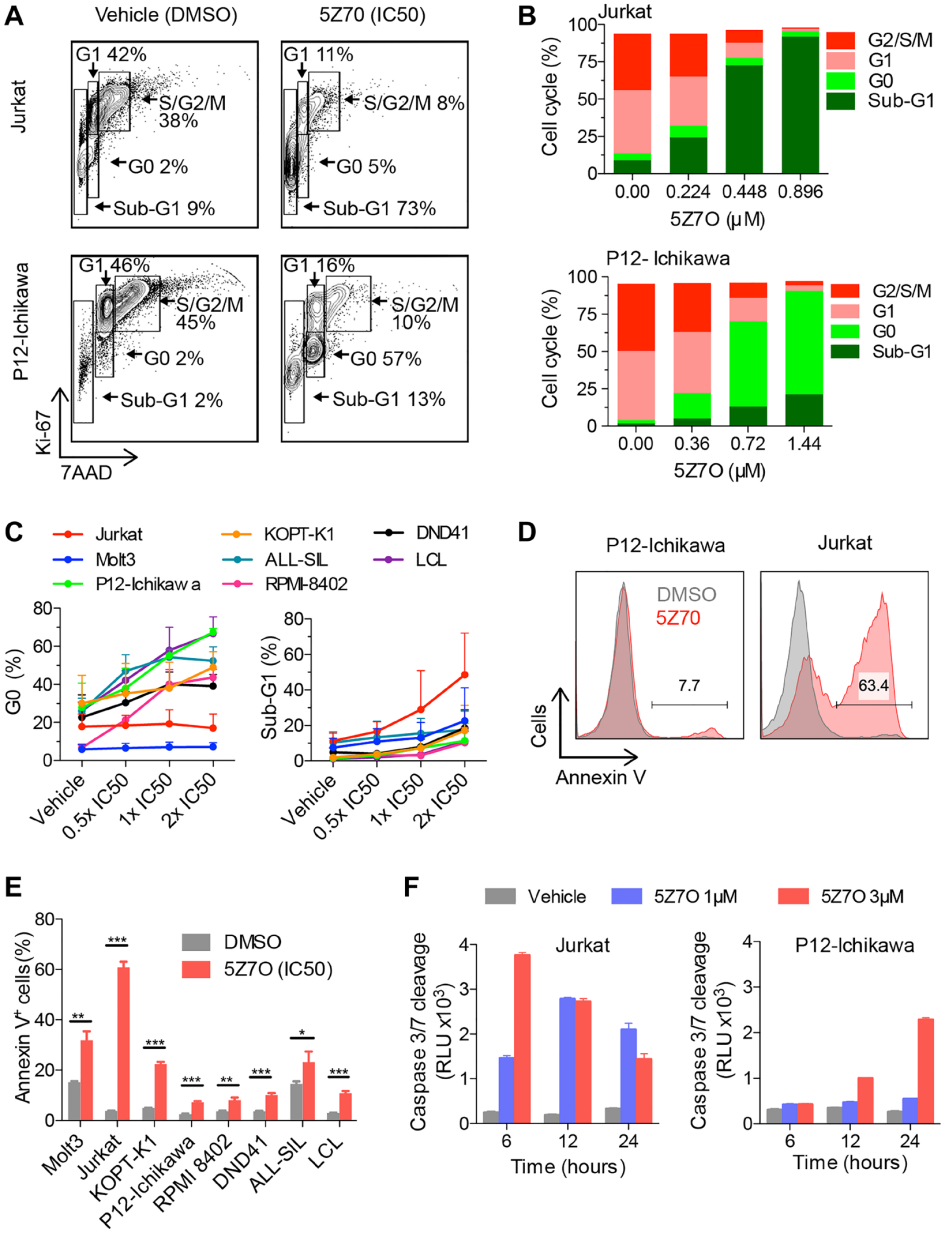
Treatment with 5Z7O inhibits cell proliferation and induces apoptosis in T-ALL cell lines. (**A**) Representative flow cytometric analysis of nuclear staining of Ki67 and 7-AAD to identify proliferating cells and DNA content, respectively. (**B**) Cell cycle distribution as described in (A) shown for Jurkat and P12-Ichikawa cell lines treated with 5Z7O for 48 hours at IC50 concentration and concentrations below and over IC50. (**C**) Percentages of cells in G0-phase and sub-G1 are shown for all cell lines. Data in (C) represents average and SEM (*n* = 3). (**D**, **E**) Flow cytometry analysis of Annexin V staining in a panel of T-ALL and non-leukemic LCL cell lines following 48 hours of incubation with 5Z7O at the IC50 concentration (*n* = 3). (**F**) Time and dose dependency of 5Z7O induced cleavage of Caspase-3/7 in Jurkat and P12-Ichikawa cell lines (*n* = 3). Basal levels of caspase 3/7 are shown for each time point. The data represent the mean and standard deviation. ^*^ *P* < 0.05, ^**^ *P* < 0.01, ^***^ *P* < 0.001 (two-tailed Student’s *t*-test).

To investigate the cause of 5Z7O induced cytotoxicity in T-ALL cell lines, we conducted cell cycle analysis by flow cytometric detection of Ki67 and 7AAD in nuclei (Figure 2A). Jurkat cells showed a dose dependent accumulation in the sub-G1 phase of the cell cycle following 5Z7O treatment for 48 hours, whereas P12-Ichikawa cells exhibited additional accumulation in the G0-phase of the cell cycle (Figure 2B). Further analysis revealed a correlation of sub-G1 content and IC50, Jurkat cells underwent rapid accumulation in sub-G1, whereas other cell lines displayed an accumulation at the G0 phase of the cell cycle (Figure 2C).

We next assessed the ensuing expression of annexin V by flow cytometry as a readout of cell death upon 5Z7O treatment (48 hours at IC50). Consistent with previous cell cycle analysis, most sensitive cell lines (Molt3, Jurkat, and KOPT-K1) showed a significant increase of annexin V positive cells while the rest of the T-ALL cell lines showed a moderate increase (Figure 2D and 2E). Next, we studied the time- and dose-dependency of 5Z7O using luminescent detection of Caspase-3 and Caspase-7 cleavage. We focused upon Jurkat (IC50: 0.45 μM) and P12-Ichikawa (IC50: 0.72 μM) cell lines to represent different responses and treated both cell lines with 1 μM 5Z7O, corresponding to IC50, and 3 μM 5Z7O (4- and 6-fold higher than the IC50) at different times. Jurkat cells showed a peak of Caspases-3/7cleavage that shifted from 12 hours with 1 μM to 6 hours with 3 μM 5Z7O (Figure 2F). This assay also showed that P12-Ichikawa cells require higher 5Z7O concentration and prolonged time to display increased cleavage of Caspases-3/7 (Figure 2F). Collectively, these data suggest that 5Z7O inhibits cell proliferation and induces apoptosis in T-ALL cell lines.

### Inhibition of the MAP2K7-JNK pathway in T-ALL cells

**Figure 3:**
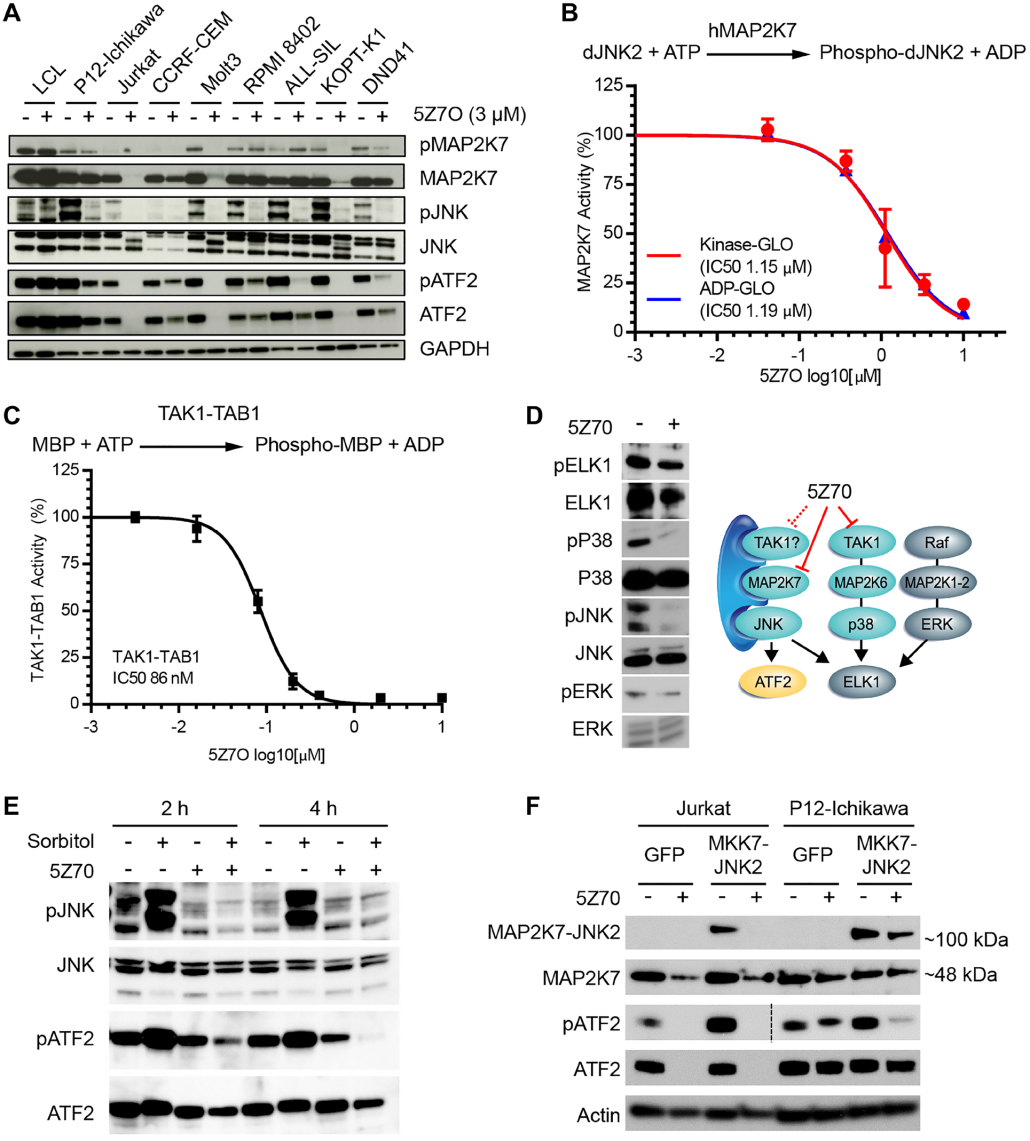
5Z7O inhibits MAP2K7 activity and downstream activation of JNK. (**A**) Immunoblot of the MAP2K7 pathway signaling in a panel of T-ALL and non-leukemic LCL cell lines following incubation with 3 μM 5Z7O for 21 hours. (**B**) Biochemical kinase assay using purified MAP2K7 protein, dead JNK2 substrate, and two methods of kinase activity based on ATP consumption or ADP production. (**C**) ADP-GLO *in vitro* kinase activity of purified TAK1-MBP protein following 30-minute pre-treatment with 5Z7O. (**D**) Immunoblot of KOPTK1 cells treated with 3 μM 5Z7O to evaluate TAK1 inhibition. Diagram indicating effects of 5Z7O in the MKK signaling pathway in T-ALL cells. (**E**) KOPTK1 cells were treated with sorbitol to evaluate capacity of 5Z7O to inhibit activated MAP2K7. (**F**) Jurkat and P12-Ichikawa cells transduced with retrovirus expressing the fusion MAP2K7-JNK2 (MKK7-JNK2) protein were treated with 5Z7O to inhibit activated MAP2K7. For phosphorylated ATF2, immunoblot in Jurkat cells required longer exposure.

To confirm that 5Z7O cytotoxicity is mediated through MAP2K7 inhibition, we assessed the MAP2K7 pathway through immunoblots using a panel of T-ALL cell lines and LCL cells as controls. For this analysis, cells were treated with 3 μM 5Z7O for 21 hours and probed for phosphorylated and total MAP2K7, JNK, and ATF2 proteins. Inhibition of MAP2K7 was evident in the reduction of downstream phosphorylation of JNK and ATF2 in most T-ALL cell lines (Figure 3A); however, phosphorylation of MAP2K7 was not significantly reduced by 5Z7O, suggesting that 5Z7O may not inhibit the kinase upstream of MAP2K7, whereas Jurkat, Molt3, and KOPTK1 cells showed a reduction of MAP2K7 protein (Figure 3A). To ascertain whether inhibition of the downstream targets JNK and ATF2 was caused by direct inhibition of MAP2K7, we next conducted *in vitro* kinase assays using purified human MAP2K7 protein and dead JNK2 as substrate and two methods assaying either ATP consumption or ADP generation. 5Z7O inhibited MAP2K7 kinase activity with an IC50 value of 1.2 μM (Figure 3B). Because 5Z7O can inhibit TAK1 and TAK1 could act as MAP3K upstream of MAP2K7 [20–22], we assessed whether 5Z7O could also inhibit TAK1 activity in a biochemical assay using TAK1-TAB1 and swine myelin basic protein (MBP) as substrate. Consistent with previous reports [22, 23], 5Z7O inhibits TAK1 activity with IC50 of 86 nM (Figure 3C), although the absence of significant reduction in phosphorylated MAP2K7 in T-ALL cells suggests that TAK1 is not the upstream kinase. However, lower levels of phosphorylated p38 and downstream ELK1 would suggest potential inhibition of TAK1 even though ELK1 can also be targeted by JNK (Figure 3D). Because we cannot rule out TAK1 inhibition in T-ALL cells, we further supported inhibition of activated MAP2K7 using two approaches. First, we showed that 5Z7O can inhibit hyperactivation of MAP2K7 induced with sorbitol (Figure 3E). Second, we retrovirally expressed the constitutively activated fusion MAP2K7-JNK2 protein in Jurkat and P12-ICHIWAKA cells, using empty retrovirus (GFP) as a control, and confirmed that 5Z7O inhibited activated MAP2K7 (Figure 3F). Altogether, 5Z7O induced cytotoxicity in T-ALL cell lines at least in part through inhibition of MAP2K7 and downstream JNK.

### Inhibition of MAP2K7 downregulates proteins of the G2/M checkpoint

**Figure 4:**
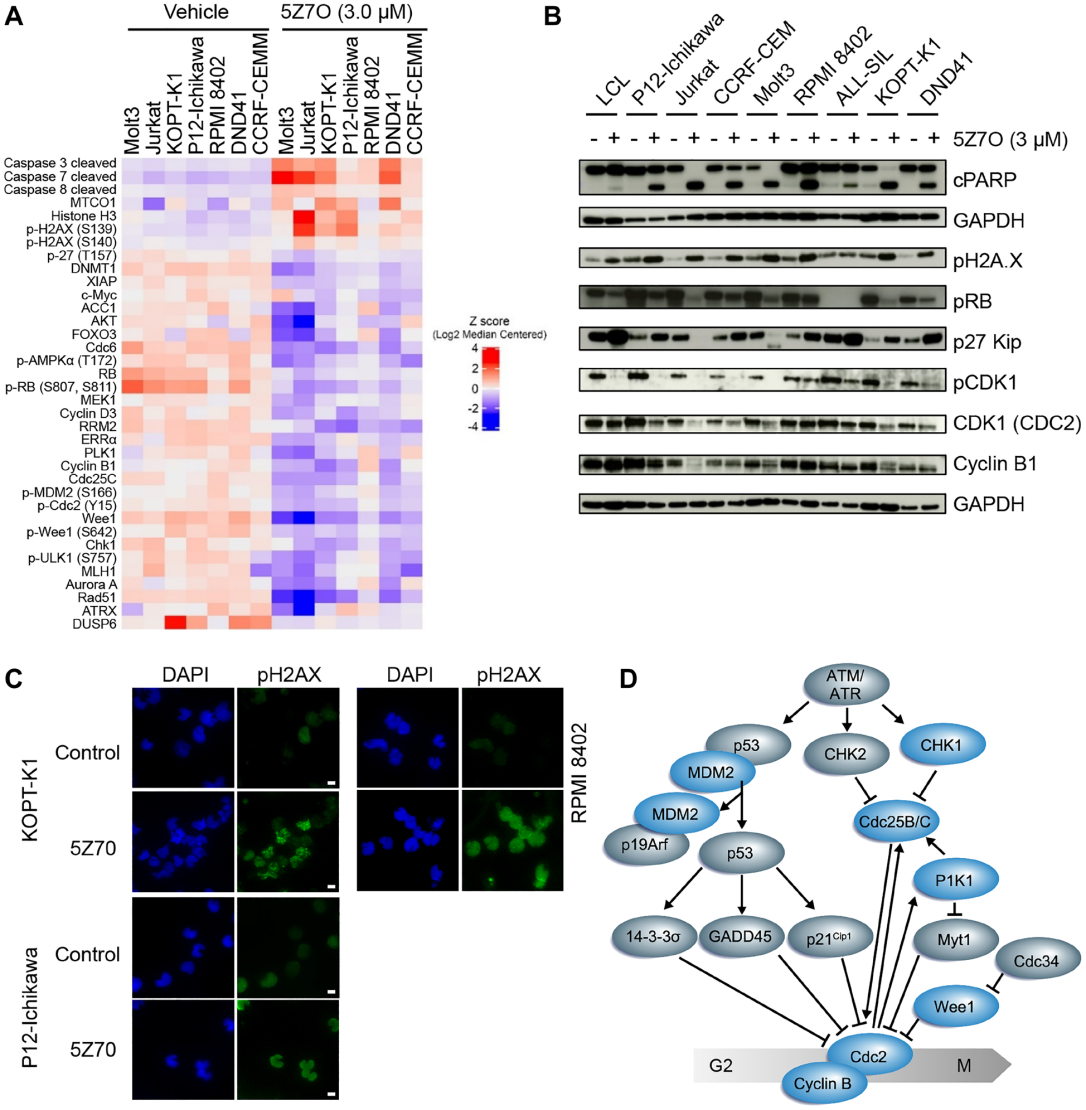
5Z7O induces activation of apoptosis pathways and disrupts cell cycle progression. (**A**) Supervised hierarchical clustering analysis of reverse-phase protein array (RPPA) performed in T-ALL cell lines treated with 3 μM 5Z7O for 21 hours. (**B**) Detection of proteins involved in apoptosis and cell cycle regulation in a panel of T-ALL and non-leukemic LCL cell lines after 5Z7O treatment as described in (A). (**C**) Immunofluorescence detection of phosphorylated H2AX (Alexa Fluor 488) in nuclei KOPT-K1, P12-Ichikawa, and RPMI8402 cells following 5Z7O treatment. DAPI was used to detect nuclei. Scale bar: 10 μm. (**D**) Diagram of DNA damage and G2/M checkpoint. Downregulated proteins upon 5Z7O treatment are indicated in blue.

Proteomic analysis of T-ALL cell lines treated with 5Z7O was conducted to identify mechanisms underlying 5Z7O-mediated cytotoxicity in T-ALL cells. Cells were incubated with either vehicle (DMSO) or 5Z7O (3 μM) for 21 hours, a condition that showed efficient inhibition of JNK phosphorylation in all T-ALL cell lines (Supplementary Figure 2), and then processed for reverse-phase protein array (RPPA). Bioinformatic analysis revealed 5Z7O induced upregulation of proteins involved in apoptosis (Caspases 3,7,8) and DNA damage (pH2AX) (Figure 4A and Supplementary Figure 3). T-ALL cells sensitive to 5Z7O, such as Jurkat and Molt3, exhibited more significant deregulation than other T-ALL cell lines. On the other hand, levels of proteins involved in cell cycle regulation were significantly reduced by 5Z7O treatment, particularly numerous critical regulators of the G2/M cell cycle checkpoint (Figure 4A). To validate RPPA results, we confirmed by immunoblots that 5Z7O induced PARP cleavage, H2AX phosphorylation, and p27 expression in T-ALL cell lines while inhibiting expression of phosphorylated CDK1 (CDC2) and Cyclin B1, both key components of the G2/M checkpoint (Figure 4B). The increased levels of phosphorylated H2AX were further confirmed in the nuclei of P12-Ichikawa, KOPT-K1, and RPMI-8402 cells by immunofluorescence (Figure 4C). Interestingly, our data is consistent with findings in *Map2k7*^−/−^ mouse embryonic fibroblasts of impaired proliferation, premature senescence, and downregulation of the G2/M cell-cycle kinase CDC2, a direct molecular target of the MAP2K7-JNK pathway (Figure 4D) [24]. However, our cell cycle analysis of T-ALL cells treated with 5Z7O did not show significant arrest at the G2/M phase of the cell cycle (Supplementary Figure 4). In addition to MAP2K7, other pathways identified as essential for T-ALL cell survival, and potential targets for drug therapy, were also inhibited by 5Z7O such as FOXO3, AKT, AMPK, and MEK [25–29].

### 5Z7O inhibits the expansion of leukemic T cells

**Figure 5:**
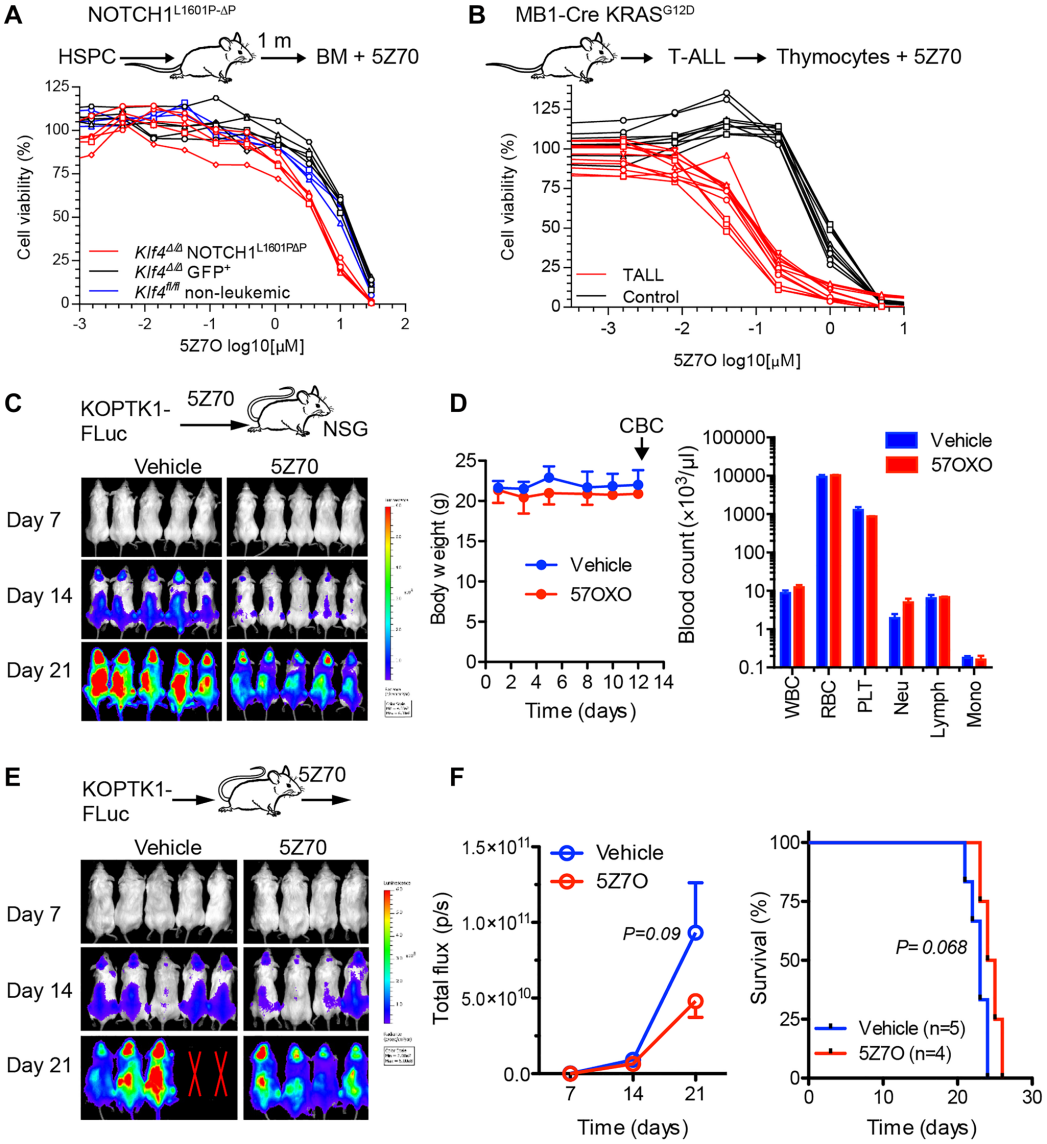
Leukemic cells from T-ALL mouse models are sensitivity to 5Z7O. (**A**) Cell viability assays of bone marrow cells from *Klf4*^∆/∆^ NOTCH1-induced T-ALL mice after 1 month of transplantation (IC50 3.9 μM, *n* = 5), non-leukemic *Klf4*^∆/∆^ bone marrow cells (IC50 11.4 μM, *n* = 5), and *Klf4*^fl/fl^ bone marrow cells (IC50 8.7 μM, *n* = 3). (**B**) Cell viability assays of thymocytes from leukemic *Kras*^LSL-G12D/+^ *.Mb1*^Cre/+^ mice (IC50 83 nM, *n* = 4) and age-matched controls (IC50 956 nM, *n* = 3). Cell viability was measured using an ATP-based assay and expressed as a percentage of the vehicle control (DMSO). IC50 values were calculated using GraphPad Prism. (**C**) KOPTK1 cells labelled with luciferase were treated *in vitro* with 5Z7O (3 μM for 3 hours) and same number of viable cells transplanted into NSG mice (*n* = 5 per group) to monitor disease progression by bioluminescence imaging (BLI). (**D**) C57BL/6 mice were treated everyday Monday to Friday for two weeks with 5Z7O (15 mg/Kg, intraperitoneal). Mice were monitored for body weight and blood counts to evaluate drug toxicity. (**E**) NSG mice (*n* = 4–5 per group) were injected with KOPTK1-luciferase cells and then administered *in vivo* with 5Z7O (15 mK/Kg). Mice were monitored by BLI. (**F**) BLI analysis and overall survival (Kaplan Meier). Data are representative of two independent experiments. *P* = 0.09 (one-tailed Student’s *t*-test).

The efficacy of 5Z7O was further evaluated using leukemic cells from two different T-ALL mouse models. First, we generated NOTCH1-induced T-ALL based on the transformation of *Klf4*^fl/fl^ *;Vav-iCre* (*Klf4*^∆/∆^) hematopoietic cells with retrovirus carrying either gain-of-function mutant *NOTCH1*^L1601P-∆P^ (leukemic) or empty retrovirus (non-leukemic) as the control followed by transplantation into cytoablated hosts [9, 30, 31]. GFP^+^ bone marrow cells from mice transplanted with *Klf4*^∆/∆^ cells transduced with empty virus showed similar cytotoxicity to bone marrow cells from *Klf4*^fl/fl^ wild-type mice, whereas bone marrow cells from mice transplanted with NOTCH1^L1601P-∆P^ *Klf4*^∆/∆^ cells showed increased sensitivity (2-3-fold lower IC50) to 5Z7O treatment (Figure 5A). These data correlate with our previous finding that loss-of-KLF4 in NOTCH1-induced T-ALL mice exhibit aberrant activation of MAP2K7 [9]. Of note, NOTCH1^L1601P-∆P^ *Klf4*^∆/∆^ bone marrow cells were collected one month after transplantation to perform the cell viability assay in parallel and therefore may contain a mixture of leukemic and polyclonal expansions with activated NOTCH1 [9]. Second, T-ALL cells from the novel *Kras*^LSL-G12D/+^ *.Mb1*^Cre/+^ mouse model that spontaneously develops T-ALL were tested against 5Z7O [32]. Thymic cells collected from mice with full-blown T-ALL showed significantly increased sensitivity to 5Z7O compared to thymic cells from healthy age-matched control mice (Figure 5B). These data suggest that T-ALL cells are more sensitive to 5Z7O irrespective of the driver mutation, which broadens potential patient application.

To evaluate whether inhibition of MAP2K7 with 5Z7O prevents leukemia cells from expanding *in vivo*, we first treated KOPTK1 cells labeled with firefly luciferase (KOPTK1-Fluc) with 5Z7O or vehicle for three hours and transplanted the same number of viable cells to NSG mice. Bioluminescence imaging (BLI) shows a reduction in the expansion of 5Z7O-treated KOPTK1 cells compare to vehicle controls (Figure 5C). Before *in vivo* treatment, we evaluated the potential toxicity of 5Z7O (15 mg/Kg) in mice through a two-week daily intraperitoneal administration, which shows no alterations neither in body weight nor blood cell counts (Figure 5D). Finally, NSG mice were injected with KOPTK1-luciferase cells and then administered with 5Z7O using the regimen described above. 5Z7O treatment showed a modest inhibition of leukemic cell expansion with a slight improvement in survival (Figure 5E, 5F). This finding may suggest that 5Z7O did not reach a therapeutic concentration to control an aggressive leukemia in this model, and therefore, more potent MAP2K7 inhibitors need to be developed for clinical translation.

### Anti-leukemic properties in human T-ALL

**Figure 6:**
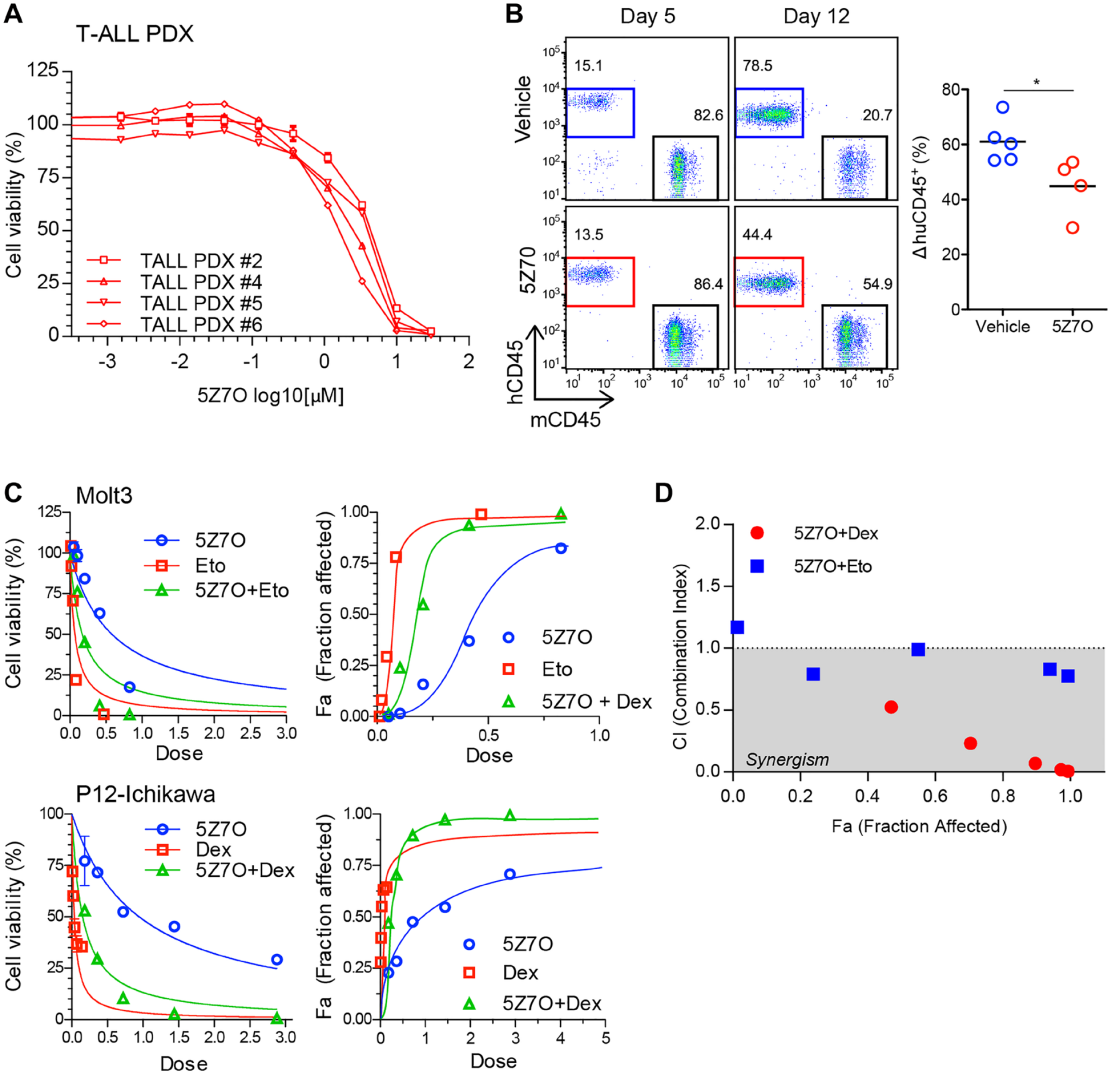
5Z7O is effective against human T-ALL PDXs and shows synergistic interaction with dexamethasone. (**A**) Cytotoxicity assays of four pediatric T-ALL PDX cells after treatment with 5Z7O for 48 hours (IC50s 1.6-4.0 μM). Cell viability was measured using an ATP-based assay and expressed as a percentage of the vehicle (DMSO) control. IC50 values were calculated using GraphPad Prism. (**B**) T-ALL PDX cells were transplanted into NSG mice (*n* = 5 per group) and treated *in vivo* with 5Z7O (15 mg/Kg daily). Flow cytometric detection of human CD45 leukemic cells after 5 and 12 days of treatment. Data is representative of two independent experiments. (**C**) Dose response curves of 5Z7O in combination with dexamethasone in the P12-Ichikawa cell line and with etoposide in the Molt3 cell line. (**D**) Plot of combination index (CI) versus fraction affected (Fa) of 5Z7O combined with dexamethasone or etoposide. CI values were determined using CompuSyn software, CI < 1.0 corresponds to synergism, CI = 1.0 to additive effect, and CI > 1.0 to antagonism. ^*^ *P* < 0.05 (two-tailed Student’s *t*-test).

To test the effect of MAP2K7 inhibition in T-ALL patient samples, we generated a panel of four T-ALL patient-derived xenograft (PDX) cells and evaluated sensitivity to 5Z7O treatment. The viability of patient T-ALL PDX cells was significantly reduced by 5Z7O with IC50 ranging from 1.6 to 4.0 μM (Figure 6A). This is particularly important because it shows that patient samples with different cytogenetics are sensitive to pharmacological inhibition of MAP2K7 with 5Z7O. Next, we transplanted a T-ALL PDX cell line into NSG mice and started treatment with 5Z7O for two weeks when engraftment was detectable in peripheral blood and found that 5Z7O controlled expansion of leukemic cells during treatment (Figure 6B), although there were no significant differences in survival after discontinuing treatment (not shown). Finally, we evaluated the anti-leukemic effect of 5Z7O in combination with dexamethasone and etoposide, two ALL chemotherapy agents. The effect of drug combination was analyzed by calculation of combination index using CompuSyn software [33]. 5Z7O showed a synergistic anti-leukemic effect with dexamethasone in the P12-Ichikawa cell line, supporting a potential use of MAP2K7 inhibitors in the induction phase (Figure 6C, 6D). Additionally, the combination of 5Z7O and etoposide produced an additive effect in Molt3 cells (Figure 6C, 6D). These findings suggest MAP2K7 inhibitors could be used in frontline, but further studies are needed to bring 5Z7O or other small molecules with inhibitory capacity of MAP2K7 to the clinic.

## DISCUSSION

Although most pediatric patients with ALL have a good prognosis and respond well to current drug therapy, T-ALL patients with the refractory and relapsed disease need alternative therapies able to eliminate chemoresistant leukemic cells with leukemia initiating capacity [1, 34]. New targeted therapies and immunotherapies with success in refractory and relapsed B-ALL have not been as promising in T-ALL patients [3]. Therefore, discovering novel molecular targets is essential to improve the outlook of children with T-ALL, particularly those with clinical features associated with the worst prognosis, by advancing the development of alternative therapies.

Our group reported a novel tumor suppression function for the transcription factor KLF4 in pediatric T-ALL [9, 13]. Both genetic loss-of-KLF4 in the NOTCH1-induced T-ALL mouse model or epigenetic KLF4 inactivation via CpG methylation in children with T-ALL caused aberrant activation of MAP2K7 and the downstream target JNK [9, 14, 35, 36]. Interestingly, in the T-ALL mouse model, this kinase pathway was also activated in the rare leukemia-initiating cell population, suggesting that inhibition of MAP2K7 could potentially eradicate leukemia-initiating cells responsible for chemoresistance and relapse. JNK inhibitors were used first to target the MAP2K7 pathway because many small molecules have been studied in clinical trials for applications in inflammatory disorders and cancer [37]. The compounds CC-401 (Phase I) and orally active AS602801 (Phase II) were able to control the expansion of T-ALL patient-derived xenograft cells both *in vitro* and *in vivo* [14, 19, 38]. However, the inhibitory activity requiring micromolar concentrations made it challenging to reach therapeutic concentrations with minimal toxicity [37].

Based on these findings, direct inhibition of MAP2K7 would be a better approach to inhibit this pathway. A recent study based on crystal structure revealed a flexible inactive state of MAP2K7 that allows binding of small molecules to the ATP-binding pocket and that MAP2K7 can be targeted with type I (active state), such as 5Z7O, and type II (inactive state) inhibitors in reversible and irreversible fashion [39]. In this work, we investigated the anti-leukemic properties of the chemical compound 5Z7O because it was shown to inhibit MAP2K7 through covalent binding to cysteine 218 located in the ATP binding pocket, and 5Z7O was identified in the Library of Integrated Network-based Cellular Signatures (LINCS) project as potential MAP2K7 inhibitor. The resorcylic lactone aromatic ring in 5Z7O resembles the adenosine ring of ATP and therefore competes with ATP binding to MAP2K7. In addition to MAP2K7, 5Z7O has been investigated as a TAK1 inhibitor in multiple disorders such as ischemic strokes, inflammatory diseases, and cancer [21, 40, 41]. Dual inhibition of MAP2K7 and TAK1 could be beneficial in T-ALL because TAK1 may act as MAP3K upstream of MAP2K7; however, lack of inhibition of MAP2K7 phosphorylation in most T-ALL cell lines suggested that 5Z7O cytotoxicity was mediated mainly through MAP2K7 inhibition. The synergism of 5Z7O with dexamethasone suggests the potential utility of this combination. While our primary interest lies in pediatric T-ALL, pharmacological MAP2K7 inhibition could have broader applications in other malignancies with aberrant MAP2K7 activation [8]. In fact, MAP2K7 transcripts are elevated in most cancer cell line types represented in the Cancer Cell Line Encyclopedia [42]. Colorectal cancer patients with a higher risk of liver metastases display low levels of miR-493 and elevated MAP2K7 expression [43]. In addition, lung cancer patients carrying a rare MAP2K7 p.Glu116Lys variant showed poor prognosis and increased metastases [44].

Although analysis of the human proteome indicates low levels of MAP2K7 expression in most normal tissues, including hematopoietic cells, it is important to highlight MAP2K7 physiological functions to gauge potential toxicities [45]. A limitation on the study of MAP2K7 has been the generation of mice with embryonic *Map2k7* gene deletion because of embryonic lethality stemming from defects in hepatocyte proliferation [24]. However, heterozygous *Map2K7* deletion retained sufficient developmental functionality to overcome E13.5 embryonic lethality and defects associated with hepatocyte organization [24]. Interestingly and similar to the effect of 5Z7O in T-ALL cell lines, *Map2k7*^−/−^ hepatoblasts showed diminished levels of the cdc2 protein involved in the G2/M phase of the cell cycle [24]. Deleting MAP2K4, another MAP2K kinase that phosphorylates JNK, results in a similar defect in hepatogenesis, indicating that JNK activity is driving liver development [46]. JNK activity is increased in immature double positive thymocytes, presumably mediated via MAP2K7, and involved in the negative selection of immature thymocytes [47]. MAP2K7 inhibition may have an anti-inflammatory role because peripheral T cells activate signaling pathways downstream of TCR, including MAP2K7. Collectively, these findings suggest pharmacological inhibition of MAP2K7 is expected to have a minimal deleterious effect on adult tissue homeostasis and warrants further pre-clinical studies.

In summary, we provide proof-of-principle data in support of targeting the MAP2K7 pathway in pediatric T-ALL. The chemical 5Z7O shows selectivity to MAP2K7 and induces dose-dependent apoptosis and deregulation of the cell cycle in murine T-ALL models, established cell lines, and human patient-derived xenografts. The low efficacy observed in *in vivo* treatment suggests that more research is needed to develop small molecules with increased potency and specificity relative to MAP2K7 and evaluate their capacity to eradicate chemoresistant leukemia-initiating cells in T-ALL patients.

## MATERIALS AND METHODS

### Chemical treatment of T-ALL cell lines

The following cell lines were cultured in RPMI-1640 medium supplemented with 10% fetal bovine serum: Molt3, Jurkat, KOPT-K1, P12-Ichikawa, DND41, RPMI-8402, ALL-SIL, CCRF-CEM, and LCL (an EBV-transformed lymphoblastoid cell line). 5Z-7-oxozeaenol (5Z7O) was purchased from Tocris (Cat. #3604), dissolved in DMSO to a stock concentration of 25 mM, and stored at −20°C. Cells were treated with different concentrations of 5Z7O for 24 and 48 hours. Cells were tested regularly for mycoplasma and authenticated using STR fingerprinting every 6 months.

### Cell viability assay

5Z7O was diluted from stock to specified concentrations in RPMI-1640 cell culture medium supplemented with 10% fetal bovine serum. The vehicle control condition contained an equivalent concentration of DMSO (<0.5%). Cell lines were plated in triplicate at a cell density of 2 × 10^4^ cells per well (96-well plate) in the presence or absence of 5Z7O (from 1.5 nM to 30 μM) for 48 hours. Cell viability was measured using CellTiter-Glo Luminescent cell viability assay. Curve fitting and statistics, including half-maximal inhibitory concentration (IC50), were calculated using Prism GraphPad nonlinear regression.

### Apoptosis and cell cycle analysis

Cells treated with a specified concentration of 5Z7O or vehicle for 48 hours and then assayed for apoptosis and cell cycle progression. Apoptosis detection was performed using PE Annexin V apoptosis detection kit (Becton-Dickinson #559763). Cells were treated with fixation/permeabilization solution kit (Becton-Dickinson #554714) before staining with anti-Ki-67 FITC (Thermo Fisher #11-5698-82) and 7AAD for cell proliferation and DNA content, respectively. Flow cytometry analysis was conducted using FACS Canto (Becton-Dickinson Bioscience) and FlowJo software (TriStar).

### Caspases-3/7 activity assay

Jurkat and P12-Ichikawa cells were plated at a cell density of 2 × 10^4^ cells per well (96-well plate) in the presence of 1 μM or 3 μM 5Z7O. At 6, 12, and 24 hours, the Caspase-GLO 3/7 Assay System (Promega G8091) was used to evaluate caspase activity resulting from 5Z7O treatment. Luminescence values corresponding to caspase-3 and caspase-7 cleavage were plotted using Prism GraphPad.

### *In vitro* kinase assay

Purified human MAP2K7 (Origene) or TAK1-TAB protein (Promega V4088) was plated with the corresponding substrate (dead JNK2 fragment for MAP2K7 and Myelin Basic Protein for TAK1) along with vehicle (DMSO) or 5Z7O. TAK1-TAB1 is used because TAK1-binding protein (TAB) is required to allow ATP binding and the fusion TAK1-TAB1 is constitutively active [48]. After preincubation for 30 minutes, ATP was added to initiate kinase activity for 30 minutes. The resulting enzymatic activity was measured through consumption of ATP (Kinase-GLO^®^ Plus Kinase Assay System, Promega #V3771) or generation of ADP (ADP-GLO, Promega #V6930). Luminescence values were calculated as the percentage of vehicle treated control. The plot, non-linear curve fit, and IC50 calculations were conducted using Prism GraphPad.

### Immunoblot analysis

After 21 hours of 5Z7O treatment at specified concentrations, cells were lysed with SDS lysis buffer containing (10 mM Tris pH 7.4 containing 1% SDS and 1 mM PMSF) and supplemented with Halt Protease and Phosphatase Inhibitor Cocktail (Thermo Fisher). Protein lysates were electrophoresed onto SDS PAGE gel before transferred to the PVDF membrane for blocking, antibody probing, and developing. Antibodies corresponding to the following target proteins were used at 1:1000 dilution: phospho-MKK7 (#4171), MKK7 (#4172), phospho-SAPK/JNK (clone 81E11) (#4668), SAPK/JNK (#9252), phospho-ATF2 (#15411), ATF2 (#9226), PARP (#9542, 1:10,000 dilution), phospho-H2A.X (#9719), phospho-RB (#3590), p27-Kip (#3686), phospho-Cdc2 (#9111), Cdc2 (#9112), and Cyclin B1 (#4138). Secondary antibodies cross-linked with HRP (anti-rabbit IgG #7074 and anti-mouse IgG #7076) were used for respective primary antibodies at concentrations 1:20,000 to 1:50,000. Protein detection was performed using West Femto Maximum Sensitivity Substrate (Thermo Fisher) and Amersham Hyperfilm ECL (GE).

### Activation of MAP2K7 in T-ALL cell lines

KOPT-K1 cells were treated with 400 mM sorbitol for 30 minutes, and then 5Z7O (10 μM) was added for 2 or 4 hours, followed by cell lysis for immunoblot analysis. Jurkat and P12-Ichikawa cells were transduced with either MSCV(IRES-GFP) retroviral vector containing the MAP2K7-JNK2 fusion protein or empty vector as a control (GFP). Transduced cells were sorted using BD FACSAria II cell sorter to purify GFP positive cells. Sorted cells were expanded and treated with vehicle or 5Z7O (3 μM) for 21 hours, followed by cell lysis for immunoblot analysis.

### Reverse-phase protein array

After 21 hours of culture with vehicle (DMSO) or 3 μM 5Z7O, T-ALL cells were submitted frozen to the MD Anderson Cancer Center’s Reverse Phase Protein Array Core where protein lysate was prepared for hybridization of arrays. ComplexHeatmap R package was used to generate heatmap [49]. Differential analysis was performed using Linear Models of Microarray Data (R Package) [50].

### Immunofluorescence

RPMI-8402, KOPT-K1, and P12-Ichikawa cells were treated with 3 μM 5Z7O for either 6 or 21 hours. Cells were spun onto slides using a cytocentrifuge and then fixed with acetone. After incubation with blocking buffer (PBS 0.3% Triton-X100 with 5% normal goat serum), phospho-histone H2A.X primary antibody (1:50 dilution, Cell Signaling #2577) was added to each slide and incubated overnight at 4°C. After washing, Alexa Fluor 488 conjugated anti-Rabbit IgG secondary antibody (1:500 dilution) was added to each slide and incubated for 2 hours. After washing, ProLong Gold antifade mounting solution with DAPI (Invitrogen #P36935) was applied to each slide before covering it with coverslip. All images were captured with ECLIPSE 90i Nikon fluorescence microscope at 100× lens with oil.

### NOTCH1 gain-of-function mutant induction of T-ALL mouse model

Bone marrow cells were obtained from femur and tibia from *Klf4*^fl/fl^ *Vav-iCre* mice four days after intraperitoneal injection of 5-fluorouracil (150 mg/kg). After 48-hour culture in X-Vivo15 supplemented with murine stem cell factor (100 ng/mL), IL-3 (6 ng/mL), IL-6 (10 ng/mL), hematopoietic stem/progenitor cells were transduced twice with retroviral supernatant containing MIGR1-NOTCH1-L1601P-∆P. Following 48 hours of culture, the transduced cells (0.5 × 10^6^ cells per mouse) were transplanted into lethally irradiated C57BL/6J mice (950 Rads). Leukemic cell expansion was monitored in peripheral blood through a flow cytometric analysis panel consisting of GFP, CD4, and CD8. Four weeks after transplantation, bone marrow was isolated from the femur of leukemic and non-leukemic mice for *in vitro* treatment with 5Z7O.

### Patient samples

T-ALL diagnostic bone marrow samples were obtained with consent from patients treated at Texas Children’s Cancer and Hematology Center under an Institutional Review Board approved protocol and transplanted (0.5–1.0 × 10^6^ cells/mouse) into 10-week-old female NSG mice that had received 200 Rads irradiation. Peripheral blood sampled from the tail vein was routinely monitored for human CD45 positive cells via flow cytometry. Human leukemic cells were collected from femur and tibia and spleen, examined for expression of human CD45 surface antigen, and either frozen or treated with 5Z7O according to cell line viability assay using X-Vivo15 media supplemented with IL-7 (10 ng/mL).

### *In vivo* studies of 5Z7O

To evaluate toxicity, 5Z7O prepared in 10% DMSO and 90% corn oil (15 mg/Kg) was administered intraperitoneally every day from Monday to Friday for two weeks and mice were monitored for body weight and complete blood counts. First, NSG mice were transplanted with KOPTK1-FLuc cells (2.5 × 10^5^) previously treated with 57Z0 (3 μM for 3 hours) and monitored for BLI. Next, NSG mice were transplanted with KOPTK-1 cells labeled with firefly luciferase (2.5 × 10^5^) and treated for three weeks with 5Z7O or vehicle using the same dose and administration plan as toxicity experiment and evaluated by bioluminescence at the end of each week. For bioluminescence detection, the images were acquired in anesthetized mice using the IVIS Imaging System (Xenogen) 10 minutes after intraperitoneal injection with 50 mg/kg D-luciferin. Finally, NSG mice were injected with T-ALL PDX cells (1 × 10^6^) and randomized a week later into two groups (administration of vehicle or 15 mg/Kg 5Z7O) and monitored for expansion of human CD45 positive cells in peripheral blood by flow cytometry.

### Statistics

All sample sizes (*n* values) indicated in each figure legend correspond to independent biological replicates. Experimental results with more than 8 values were confirmed to follow normal distribution by the D’Agostino-Pearson normality test, and no significant differences in the variance between groups were detected using the F test (Prism Graphpad). Unpaired two-tailed Student’s *t*-test was used for statistical analysis. *P* values were determined using GraphPad software. Results with a *P* value <0.05 were considered statistically significant.

## SUPPLEMENTARY MATERIALS



## Abbreviations

T-ALL: T-cell acute lymphoblastic leukemia
KLF4: Krüppel-like factor 4
MAP2K7: mitogen-activated kinase kinase 7
MAP2K4: mitogen-activated kinase kinase 4
5Z7O: 5Z-7-Oxozeaenol
JNK: c-Jun N-terminal kinase
BLI: bioluminescence imaging
NSG: NOD scid gamma mouse
PDX: patient-derived xenograft
RPPA: reverse phase protein array
ATF2: activating transcription factor 2
