# Supplementary material for: Inhibition of the MAP2K7-JNK pathway with 5Z-7-oxozeaenol induces apoptosis in T-cell acute lymphoblastic leukemia
Source: Oncotarget. 2021 Aug 31;12(18):1787–801. doi: 10.18632/oncotarget.28040 (PMC8416565; doi:10.18632/oncotarget.28040)
Supplement: Supplementary file 1 [file oncotarget-12-1787-s001.pdf]

Inhibition of the MAP2K7-JNK pathway with 5Z-7-oxozeaenol induces apoptosis in T-cell acute lymphoblastic leukemia

SUPPLEMENTARY MATERIALS

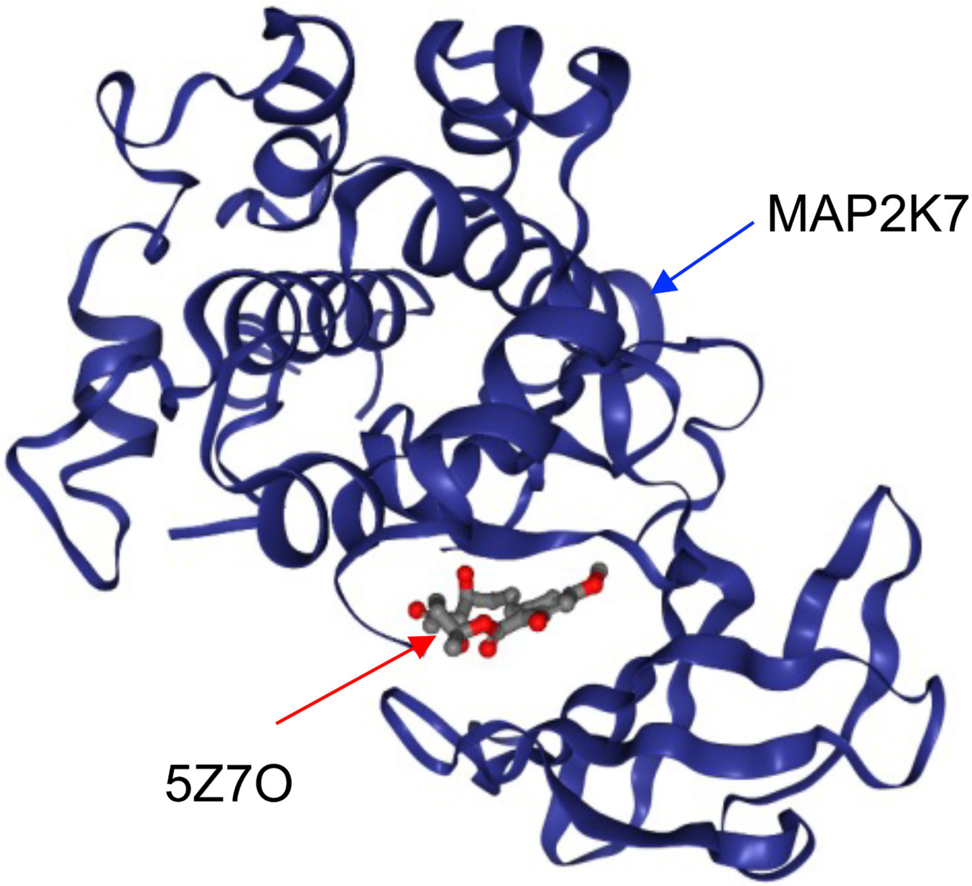

Supplementary Figure 1: 5Z-7-oxozeaenol (5Z7O in red) binding location in the ATP binding pocket of MAP2K7 (in blue).

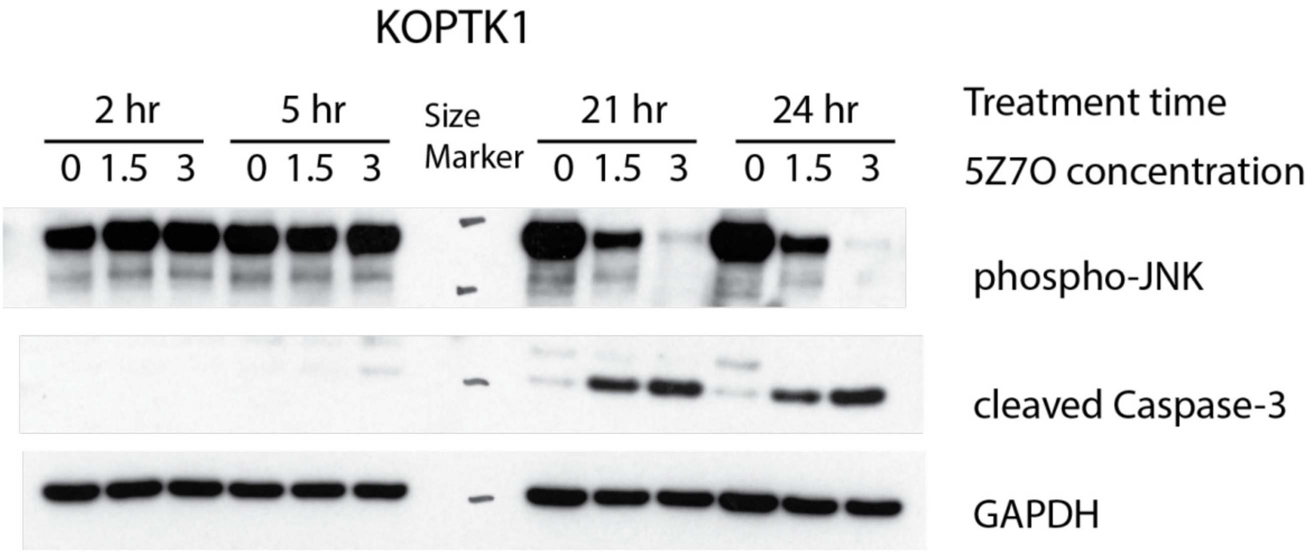

Supplementary Figure 2: Inhibition of phospho-JNK and increased caspase-3 cleavage over 24-hour treatment with 1.5 and 3 µM 5Z7O.

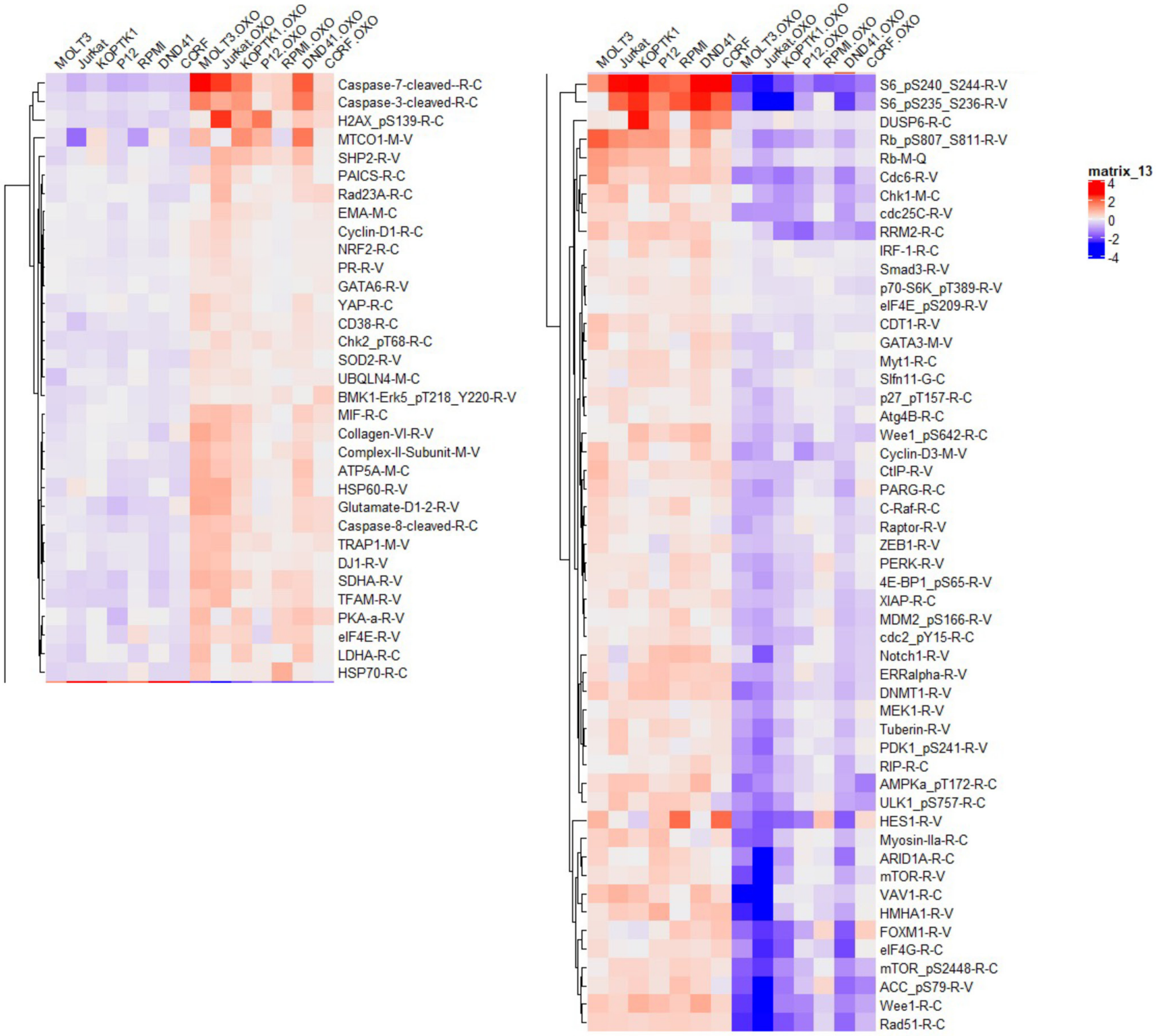

Supplementary Figure 3: Unsupervised heat map of 85 proteins with significant difference (*p*-value < 0.05) in protein expression following 5Z7O treatment in a panel of T-ALL cell lines.

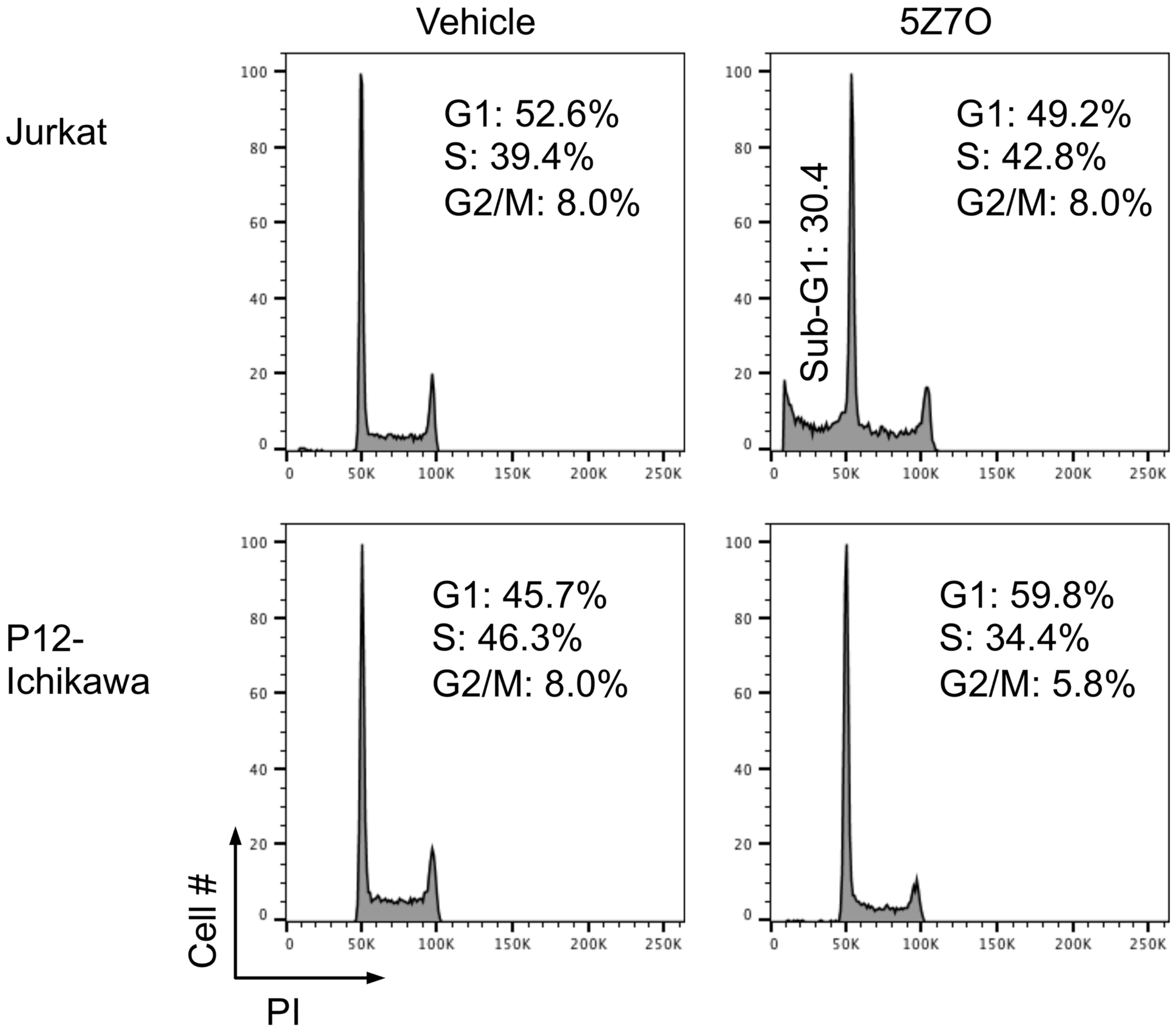

Supplementary Figure 4: DNA content analysis of JURKAT and P12-ICHIWAKA cells incubated with 0.45 µM (JURKAT) and 0.72 µM (P12-ICHIKAWA) 5Z7O for 48 hours. Flow cytometric analysis of propidium iodine (PI) staining.
